# High *RAS* Allele Frequency Signals Increased Risk of *TERT* Promoter Mutations in Thyroid Tumors

**DOI:** 10.3390/cancers17172851

**Published:** 2025-08-30

**Authors:** Coralie Lefebvre, Hannah Greenspoon, Kayla E. Payne, Emily Steinberg, Felicia Tewfik, Gianluca Savoia, Sabrina Daniela da Silva, Marc Pusztaszeri, Véronique-Isabelle Forest, Richard J. Payne

**Affiliations:** 1 Faculty of Medicine, McGill University, Montreal, QC H3A 2M7, Canada; 2 Faculty of Arts and Science, Queen’s University, Kingston, ON K7L 3N6, Canada; 3 Faculty of Arts, McGill University, Montreal, QC H3A 2M7, Canada; 4 Department of Family Medicine, University of Ottawa, Ottawa, ON K1N 6N5, Canada; 5 Faculty of Medicine, Université de Montréal, Montreal, QC H3T 1J4, Canada; 6 Department of Otolaryngology—Head and Neck Surgery, Sir Mortimer B. Davis—Jewish General Hospital, McGill University, Montreal, QC H3T 1E2, Canada; 7 Department of Pathology, Sir Mortimer B. Davis—Jewish General Hospital, McGill University, Montreal, QC H3T 1E2, Canada; 8 Department of Otolaryngology—Head and Neck Surgery, Royal Victoria Hospital, McGill University, Montreal, QC H4A 3J1, Canada

**Keywords:** thyroid cancer, molecular mutations, molecular testing, *RAS* mutation, *TERT* mutation, allele frequency, aggressiveness

## Abstract

Background/Objectives: *RAS* mutations are among the most common genetic alterations in thyroid cancer and are generally associated with less aggressive behavior. However, when co-occurring with *TERT* (telomerase reverse transcriptase) promoter mutations, known markers of poor prognosis, tumors exhibit markedly more aggressive features. The allele frequency (AF) of *RAS* may serve as a potential indicator of clonal dominance and the likelihood of additional high-risk mutations, such as *TERT* mutation. This study aims to assess whether a high *RAS* AF correlates with the presence of coexisting *TERT* promoter mutations and other molecular alterations. Methods: A retrospective chart review was performed on 111 patients with thyroid nodules harboring *RAS* mutations, either alone or in combination with *TERT* promoter mutations. All patients underwent molecular testing with ThyroSeq v3 and subsequent thyroidectomy at McGill University teaching hospitals. *RAS* AF was analyzed in relation to *TERT* mutation status, nodule size, and other molecular alterations including copy number alterations (CNA) and gene expression profiles (GEP). Results: The mean *RAS* AF was significantly higher in nodules with both *RAS* and *TERT* mutations (38.1%) compared to those with *RAS* mutations alone (22.1%) (*p* = 0.002). Nodules with coexisting *TERT* mutations were also significantly larger (mean size: 3.7 cm vs. 2.4 cm; *p* = 0.005). Malignant nodules, regardless of *TERT* status, showed a trend toward higher *RAS* AF than benign nodules (23.0% vs. 16.3%; *p* = 0.052). Higher *RAS* AF was also associated with the presence of CNA and/or GEP positivity. Notably, GEP was positive in 100% of nodules with both *RAS* and *TERT* mutations, compared to 37.5% in *RAS*-only nodules (*p* = 0.002). Conclusions: A high *RAS* AF increases the likelihood of a *TERT* promoter mutation and other genetic alterations, highlighting the importance of *RAS* AF in optimizing patient care and management.

## Simple Summary

Thyroid cancer is the most common endocrine malignancy. When a thyroid nodule is found to be indeterminate on biopsy, despite the fact that the majority are benign, surgery is often required for definitive diagnosis. To reduce unnecessary surgical interventions, accurate pre-operative risk assessment is critical. Molecular testing plays an increasingly important role by identifying oncogenic mutations and providing insights into their potential malignancy and aggressiveness. *RAS* mutations are among the most commonly found genetic changes in thyroid nodules and are generally associated with predictable clinical behavior. In contrast, *TERT* (telomerase reverse transcriptase) promoter mutations in conjunction with a *RAS* mutation are strongly associated with tumor aggressiveness, recurrence, and poor prognosis. The allele frequency (AF), representing the proportion of mutated DNA within a sample, has been correlated with tumor behavior in certain mutations, such as *BRAF V600E*. However, its prognostic significance in *RAS*-mutated tumors remains unclear. This study investigates whether a high *RAS* AF can serve as a predictive marker for coexisting *TERT* promoter mutations. Identifying such a correlation could improve pre-operative risk stratification and support more personalized approaches to thyroid cancer management.

## 1. Introduction

Thyroid cancer is the most common endocrine malignancy and ranks ninth in overall cancer incidence globally, with a rising prevalence, particularly among women [1]. It is diagnosed at an annual rate of approximately 13.5 cases per 100,000 individuals, while the mortality rate remains low, at 0.5 deaths per 100,000 per year [2]. The biological behavior of thyroid cancer varies widely, ranging from indolent to highly aggressive forms, which can complicate treatment planning [3]. Given the relatively low mortality associated with thyroid cancer, accurate preoperative assessment of thyroid nodules is essential to guide clinical decision-making and optimize patient outcomes. It helps avoid unnecessary surgical interventions that may result in postoperative complications and lifelong thyroid hormone replacement [4].

Molecular testing is an advanced diagnostic tool used to evaluate thyroid nodule samples for genetic alterations, such as point mutations, copy number alterations (CNA) and gene expression profile (GEP) which can serve as markers of benignity or malignancy [5,6]. Among the most clinically relevant mutations, *BRAF V600E* and *TERT* promoter mutations are strongly associated with malignancy and aggressive tumor behavior [7,8]. The *TERT* gene plays a key role in maintaining chromosomal integrity by preventing telomere shortening. While normally silent in most adult tissues, mutations in the *TERT* promoter can lead to aberrant gene activation, promoting unchecked cellular proliferation and contributing to tumor progression and aggressiveness [9]. In contrast, *RAS* genes encode signaling molecules involved in regulating cell growth, differentiation, and survival. Activating point mutations in *RAS* can result in persistent downstream pathway activation, driving tumorigenesis [10]. However, *RAS*-mutant thyroid tumors typically exhibit a more indolent clinical course compared to those harboring *BRAF V600E* or *TERT* mutations [11]. Despite this, *RAS* mutations are associated with a wide range of malignancy risk, estimated between 37% and 85%, which adds complexity to clinical decision-making and underscores the need for additional risk stratification tools [12].

In the context of molecular testing, allele frequency (AF) refers to the percentage of mutant DNA detected within a collection of cells examined [13]. This parameter helps estimate the degree of intratumoral genetic heterogeneity and how extensively a mutation is distributed across the tumor cell population. Elevated AF values typically indicate that the mutation is present in a large fraction of tumor cells, whereas lower values are more consistent with subclonal alterations confined to a smaller subset of cells [14].

Recent studies have also proposed AF as a potential substitute marker of tumor burden, with increasing AF values possibly associated with more aggressive clinical behavior and poor prognosis [15]. Indeed, AF has recently helped in the identification of aggressive thyroid cancer in the case of certain mutations [16]. For example, studies have shown that an AF higher than 25.8% for a *BRAF V600E* mutation is indicative of a higher level of invasiveness and aggressiveness [17]. However, in the case of *RAS* mutations, AF studies have not demonstrated a direct correlation between higher AF and increased tumor aggressiveness. A recent study has postulated that a higher *RAS* AF may increase the likelihood of coexisting genetic alterations, such CNA and GEP [12]. We therefore hypothesized that a high *RAS* AF could similarly be associated with a second high-risk mutation, such as a *TERT* promoter mutation, potentially contributing to more aggressive tumor behavior. Identifying a potential *RAS* AF threshold predictive of such co-mutations could improve preoperative risk stratification and guide surgical decision-making. This study aims to assess the extent to which a high *RAS* mutation AF correlates with the presence of a *TERT* promoter mutation.

## 2. Materials and Methods

### 2.1. Patient Samples

A retrospective chart review of 362 surgical patients was conducted at two McGill University teaching hospitals in Montreal, Canada, between January 2021 and March 2025. All selected patients were over 18 years of age, had undergone thyroid surgery, and had molecular testing using ThyroSeq v3. ThyroSeq v3 is a next-generation sequencing-based multigene panel for thyroid cancer used for identifying different alterations, including point mutations, insertions, deletions, gene fusions, CNA and GEP. This molecular test analyzes more than 112 genes, including *BRAF*, *RAS*, *TERT*, *RET*, and many others. Testing was performed on samples from fine-needle aspiration pre-surgery following the manufacturer’s protocol [18].

Patients were included in the study if their thyroid nodules demonstrated a *RAS*-like mutation. Molecular testing results for eligible patients included *RAS* mutation AF and other genetic alterations, such as *TERT*, CNA, and GEP. Patients with a *RAS* mutation in combination with a co-mutation other than *TERT* were excluded from the study.

Ethics approval was obtained by the Research Ethics Committee of the integrated Health and Social Services Network for West-Central Montreal 29 August 2023 (#37-2024-9661).

### 2.2. Data Collection

Out of 362 patient records reviewed, 111 met the inclusion criteria outlined above and were included in the study. Patients were categorized into two groups: those with a *RAS* mutation only, and those with both *RAS* and *TERT* promoter mutations. Baseline clinical and cytological data were collected, including patient age, sex, nodule size, and Bethesda category. Molecular data were retrieved from the ThyroSeq v3 results, including *RAS* AF, presence of *TERT* promoter mutations, GEP, CNA and the specific isoform pattern of *RAS*. Final surgical pathology reports were reviewed to collect histopathological findings and classify malignancy subtypes.

### 2.3. Statistical Analysis

Descriptive analyses were conducted to summarize baseline characteristics. Associations between *RAS* AF and other genetic alterations, including *TERT,* CNA, and GEP, were assessed. Additional correlations were examined in relation to malignancy status, Bethesda category, nodule size, and cancer types and subtypes. Statistical significance between groups was established as *p*-value ≤ 0.05 using a logistic regression model with a reported confidence interval of 95%. Associations between categorical variables were assessed using the chi-square test or Fisher’s exact test, as appropriate. Analysis of variance (ANOVA) was used for comparisons involving continuous variables. The analyses were performed using the statistical software package STATA-13 (STATA Corporation, College Station, TX, USA) and IBM Statistical Package for Social Sciences (SPSS), Version 29.0.1.0 (IBM Corp., Armonk, NY, USA).

## 3. Results

Of the 362 patient files who underwent both a ThyroSeq v3 and a thyroidectomy at one of the McGill University centers, a total of 111 patients fit the above-mentioned criteria. In addition, 7 patients both had *TERT* and *RAS* mutations and 104 patients only had *RAS* mutations. The average age for patients with both *TERT* and *RAS* mutations was 56.4 years old and 49.8 years old for patients with only a *RAS* mutation. Of the patients with *TERT* and *RAS* mutations, 5 (71.4%) were female while 2 (28.6%) were male. Regarding the patients with only *RAS* mutations, 86 (82.7%) were female while 18 (17.3%) were male. Therefore, there were no major differences in age and sex between the two categories of patients.

Regarding the nodule size, the patients with *TERT* and *RAS* mutations had a significantly higher size at 3.7 cm compared to 2.4 cm for patients with only a *RAS* mutation (*p* = 0.005) (Table 1).

Regarding Bethesda categories, although not statistically significant, there was a trend where patients with *TERT* and *RAS* mutations were most often associated with Bethesda IV, while patients with only a *RAS* mutation were most often linked to Bethesda III. The average *RAS* mutation AF in the cases where *TERT* mutation was present was significantly higher (at 38.1%) than the patients without *TERT* mutation (at 22.1%) (*p* = 0.002). In addition, all the patients with *TERT* and *RAS* mutations had malignant nodules, while 89 (85.6%) patients with only *RAS* mutation had malignant nodules (Table 2).

**Figure 1:**
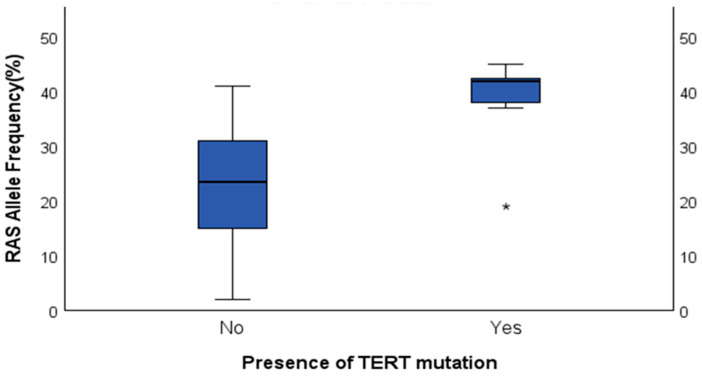
Boxplot comparing *RAS* mutation AF in thyroid nodules with and without *TERT* promoter mutations. The plot illustrates the distribution of *RAS* AF values, highlighting potential differences between *TERT*-mutant and *TERT*-wildtype groups. Boxes represent the interquartile range (IQR), with the median shown as a horizontal line; whiskers indicate 1.5 × IQR. Outliers are shown as *.

Figure 1 highlights that *RAS* AF is significantly higher in nodules with *TERT* mutations, clustering from 37% to 45%, compared to a much wider range of AF with a median around 25% in the *RAS*-only nodules. One outlier in the *TERT* and *RAS* category can be seen with an AF of 19%.

The presence of GEP was significantly higher in patients with both *TERT* and *RAS* mutations with all (100%) patients compared to 39 patients (37.5%) for those with only *RAS* mutations (*p* = 0.002). On the contrary, CNA did not seem to be associated with one category over the other, with 2 (28.6%) and 22 (21.2%) positive CNA in patients with and without a *TERT* mutation, respectively (Table 3).

Analysis of *RAS* mutation AF based on the presence of GEP and/or CNA revealed distinct trends. No comparison was possible for GEP status in *TERT* and *RAS* co-mutated nodules, since all cases were GEP-positive. However, among *RAS*-only nodules, higher *RAS* AF appeared associated with GEP positivity (ANOVA with post hoc *p* = 0.202).

CNA presence was associated with higher *RAS* AF in both groups. In *TERT* and *RAS* co-mutated nodules, the mean AF was 43.5% with CNA compared to 36.0% without, although this trend did not reach significance (ANOVA with post hoc *p* = 0.792). In *RAS*-only nodules, the difference was more pronounced: 30.1% with CNA compared to 19.9% without (ANOVA with post hoc *p* < 0.001).

Regarding malignancy, because all *TERT* and *RAS* co-mutated nodules were malignant, no further comparison could be made. However, among *RAS*-only nodules, *RAS* AF was higher in malignant (23.0%) compared to benign (16.3%) cases. ANOVA showed a significant overall difference (*p* < 0.001), with a borderline post hoc *p*-value of 0.052 between these two groups.

Finally, we observed a statistically significant increase in *RAS* AF at 25% for *NRAS* compared to 21.4% for *HRAS* and 16.6% for *KRAS* (Table 4).

Regarding thyroid cancer types, we observed a trend, although not statistically significant, where follicular thyroid carcinoma (FTC) had a higher AF at 30.8% compared to papillary thyroid carcinoma (PTC) with an AF of 22.5%, regardless of the presence of *TERT* (*p* = 0.104) (Table 5).

Although no statistically significant results were found regarding AF across malignancy subtypes, the FTC subtypes tended to have a higher *RAS* AF compared to PTC subtypes.

When evaluating the final pathology results (Table 6), *RAS*-only nodules demonstrated a higher *RAS* AF in classical and the solid/trabecular subtypes. Conversely, papillary microcarcinoma, oncocytic subtype and benign follicular nodular disease categories had the lowest *RAS* AF. With respect to the final pathology of the *RAS* and *TERT*-positive nodules, the highest *RAS* AF, at 45.0%, corresponded to a widely invasive oncocytic carcinoma with a poorly differentiated component. Two cases had a *RAS* AF of 42.0% and were, respectively, a high-grade widely invasive FTC and a differentiated high-grade follicular variant of papillary thyroid carcinoma (FVPTC). The two cases with both *TERT* and *RAS* mutations that had the lowest *RAS* AF, averaging 28.0%, were classified as minimally invasive FTC.

## 4. Discussion

In recent years, molecular testing has allowed thyroid specialists to gather more information on the type of mutations present in PTC and FTC, and *RAS*-like mutations are one of the most common [3]. In addition, *RAS* mutations can be present in combination with another mutation, such as a *TERT* promoter mutation. It has been demonstrated that the effect of the *TERT* co-mutation induces further PTC/FTC progression and distant metastasis [19]. Indeed, studies have shown that *RAS* with *TERT* promoter co-mutation may play a synergistic role in thyroid tumorigenesis and increase aggressiveness behavior and poor prognosis [9]. Therefore, understanding the malignancy and aggressiveness of *RAS* mutations is essential to enhance clinical decisions regarding *RAS* mutations.

The present study examined the relationship between *RAS* mutation AF and the presence of a *TERT* promoter mutation and additional genetic alterations such as GEP and CNA. One of the most important findings of our study was the significant difference in *RAS* mutation AF between patients with and without a *TERT* promoter mutation (*p* = 0.002). The average *RAS* AF was notably higher in the *RAS* and *TERT* group (38.1%) compared to the *RAS*-only group (22.1%).

Additionally, we observed that in our sample, all cases with a *RAS* AF of 42% or higher had both *RAS* and *TERT* mutations and exhibited aggressive histopathological subtypes. The highest *RAS* AF observed in the *RAS*-only group was 41%. While this suggests that a *RAS* AF above 41% may be associated with an increased likelihood of a coexisting *TERT* promoter mutation, we did not formally define a cutoff point due to limited sample size. These observations are exploratory in nature and a larger cohort would be required to establish a statistically robust threshold.

Nonetheless, this finding shows that *RAS* AF may play an important role in determining the likelihood of having a second genetic alteration, such as a *TERT* promoter mutation, which leads to more aggressive disease [12]. Identification of *RAS* mutation AF is therefore essential for creating an optimal treatment plan.

In addition, the size of the nodules was found to be significantly higher in the case of patients with both *RAS* and *TERT* mutations compared to patients with only *RAS* mutations (*p* = 0.005). In accordance with our findings, the *TERT* promoter mutation has been previously shown to correlate with larger nodule size in other carcinomas such as anaplastic thyroid carcinoma (ATC) [20]. This finding could also potentially explain the high *RAS* mutation AF in patients with *TERT* promoter mutations. Previous studies have suggested a positive correlation between increased AF or mutational load and increased tumor size [21,22]. The relationship could also be explained by the length of time the thyroid nodule had to develop, which could lead to a bigger size, a higher AF and a higher likelihood of a second genetic alteration, such as a *TERT* promoter mutation.

In keeping with the literature, we have found that an increase in the *RAS* AF led to a higher likelihood of having other genetic alterations, such as CNA and GEP [12]. Regardless of the presence of *TERT*, the *RAS* AF was higher when CNA and GEP were positive compared to when they were not. In fact, all patients with both *RAS* and *TERT* mutation were also GEP-positive compared to only 37.5% of *RAS*-only nodules. This could indicate the potential use of GEP in defining the likelihood of aggressive features and malignancy. Furthermore, this finding demonstrates that the higher the *RAS* mutation AF, the higher the likelihood of having additional genetic alterations. Moreover, we compared the differences in *RAS* AF between *HRAS*, *NRAS* and *KRAS*. We observed that *NRAS* had a significantly higher AF compared to *HRAS* and *KRAS* (*p* = 0.018).

As previously discussed, an outlier was observed in the *TERT* and *RAS* category, with a *RAS* mutation AF of 19%. This could be partially explained by differences in histopathological subtypes. Indeed, a subset of *TERT* and *RAS*-positive nodules were diagnosed as minimally invasive FTCs, which by definition lack aggressive features [23]. These nodules also exhibited lower *RAS* AF compared to *TERT* and *RAS* cases with aggressive histopathological subtypes, such as widely invasive FTC and differentiated high-grade FVPTC [24,25]. This observation underscores furthermore the potential relevance of *RAS* AF in clinical decision-making. Although *TERT* promoter mutations are generally associated with adverse outcomes, cases with low *RAS* AF did not display aggressive histology on final surgical pathology.

On the other hand, although not statistically significant, a relationship between malignancies and *RAS* AF was established. Indeed, the average *RAS* AF was higher when the final surgical pathology was malignant compared to when it was benign. This finding could furthermore help thyroid specialists to guide their treatment plan according to the *RAS* AF.

In addition to the physical manifestations of malignancy, there are specific quality of life (QOL) issues associated with having a *TERT* mutation. According to Richard Lazarus’ stress appraisal theory [26], stress is determined by the way individuals perceive and evaluate the event. The primary appraisal is the initial stage, which is the individual’s perception of the nature and significance of the event, while the secondary appraisal refers to an individual’s evaluation of their ability to cope with the perceived threat. Accordingly, this model suggests that individuals experience heightened anxiety when they appraise a situation as threatening, such as having a *TERT* mutation, and that they lack the coping skills to deal with it. Moreover, the information processing view [27] asserts that knowledge about a situation reduces stress. As a result, knowing the *TERT* status prior to surgery may lead to less overall stress with an improved QOL.

The main limitations of this study include its retrospective nature and the relatively small sample size. *TERT* promoter mutations are rare mutations in thyroid cancer, which inherently limits the number of available cases for analysis. Not only are *TERT* mutations uncommon, but their co-occurrence with *RAS* mutations is even rarer, as demonstrated in prior studies reporting very few such cases (1 case out of 145, 4 cases out of 243) [28,29]. In our cohort, only seven cases had both *TERT* and *RAS* mutations, compared with 104 cases with *RAS* mutations alone. This imbalance with the exclusion of some cases due to conflicting postoperative findings, reduced the statistical power and precision of subgroup analyses. Therefore, our results should be interpreted with caution and considered hypothesis-generating rather than definitive. However, our findings are consistent with prior studies demonstrating the clinical relevance of *TERT* mutations, and they underscore the need for larger, multi-institutional cohorts to validate these associations. In addition, the data was collected in one region, leading to geographical bias. Some patients needed to pay out of pocket for the molecular testing, which can lead to demographic bias. In addition, not every molecular testing company indicates additional information such as the AF, CNA and the GEP. Since that data was essential for conducting this research, only the patients who received a ThyroSeq V3 molecular test were admissible. This could introduce biases since not all patients with *RAS* mutations could be included in this study. In addition, tumor purity and CNA are known to influence the sensitivity of molecular testing and can significantly alter the reported AF [30,31]. However, in ThyroSeq v3, specimen cellularity is provided only as a binary designation (adequate vs. inadequate), and in our cohort all specimens were reported as adequate. Similarly, CNA is reported only as positive or negative, without quantitative information on copy number gains or losses. Therefore, our study is limited by the inability to more precisely account for tumor content or CNA complexity, which may impact the interpretation of molecular results. Finally, the study only includes surgical patients and therefore not all *RAS* patients could be included.

## 5. Conclusions

This study indicates that a higher *RAS* AF is significantly associated with the presence of coexisting *TERT* promoter mutations. These findings suggest that *RAS* AF may serve as a surrogate marker for identifying thyroid nodules with more complex and potentially aggressive molecular profiles, particularly valuable when molecular testing platforms do not assess for *TERT* mutation.

Thyroid nodules with both *RAS* and *TERT* mutations were significantly larger, had higher *RAS* AF, and showed a strong correlation with positive GEP results compared to nodules with *RAS* mutations alone. Incorporating *RAS* AF into the molecular evaluation of thyroid nodules may improve the prediction of high-risk co-mutations, thereby improving individualized management and optimizing patient care and QOL.

## Figures and Tables

**Table 1:** Baseline characteristics of 111 thyroid nodules with *RAS*-only mutation or *RAS* and *TERT* co-mutation.

Variant	*RAS + TERT* Co-Mutation (%) (n = 7)	*RAS* Mutation Only (%) (n = 104)	*p*-Value
Age (years)			
mean ± SD * (CI)	56.4 ± 9.4 (95% CI: 47.8–65.1)	49.8 ± 13.2 (95% CI: 47.2–52.4)	0.118
Sex			
Female	5 (71.4)	86 (82.7)	0.607
Male	2 (28.6)	18 (17.3)	
Nodule size (cm)			
mean ± SD * (CI)	3.7 ± 1.1 (95% CI: 2.6–4.7)	2.4 ± 1.1 (95% CI: 2.2–2.6)	0.005

* SD: standard deviation, CI: confidence interval, cm: centimeter.

**Table 2:** Comparison of clinical and molecular features between *RAS*-only mutation or *RAS* and *TERT* co-mutation.

Variant	*RAS + TERT* Co-Mutation (%) (n = 7)	*RAS* Mutation Only (%) (n = 104)	*p*-Value
Bethesda Category			0.065
Bethesda III	2 (28.6)	55 (52.9)	
Bethesda IV	5 (71.4)	31 (29.8)	
Bethesda V	0 (0)	18 (17.3)	
AF (%) of *RAS* Mean ± SD	38.1 ± 8.8	22.1 ± 10.6	0.002
Pathology			0.591
Benign	0 (0)	15 (14.4)	
Malignant/NIFTP *	7 (100.0)	89 (85.6)	

* NIFTP: Non-invasive follicular thyroid neoplasm with papillary-like nuclear features, AF: allele frequency, SD: standard deviation.

**Table 3:** Association between *RAS* mutation and second molecular alterations including CNA and GEP.

Molecular Alterations	*RAS + TERT* Co-Mutation (%) (n = 7)	*RAS* Mutation Only (%) (n = 104)	*p*-Value
GEP(%)			0.002
Yes	7 (100.0)	39 (37.5)	
No	0 (0)	65 (62.5)	
CNA(%)			0.643
Yes	2 (28.6)	22 (21.2)	
No	5 (71.4)	82 (78.8)	

**Table 4:** Association between molecular alterations, malignancy, isoform patterns and *RAS* AF for patients with a *RAS* mutation.

Groups	Average *RAS* AF (%)	Standard Deviation (SD)	n	*p*-Value (ANOVA)
*TERT* and GEP comparison				<0.001
*TERT*+/GEP+	38.1	8.8	7	
*TERT*+/GEP−	-	-	0	
*TERT*−/GEP+	24.3	9.2	39	
*TERT*−/GEP−	20.7	11.1	65	
*TERT* and CNA comparison				<0.001
*TERT*+/CNA+	43.5	2.1	2	
*TERT*+/CNA−	36.0	9.8	5	
*TERT*−/CNA+	30.1	6.9	22	
*TERT*−/CNA−	19.9	10.4	82	
Pathology and *TERT*				<0.001
*TERT*+/Benign	-	-	0	
*TERT*+/Malignant	38.1	8.8	7	
*TERT*−/Benign	16.3	10.5	15	
*TERT*−/Malignant	23.0	10.3	89	
Isoform pattern of *RAS*				0.018
*HRAS*	21.4	10.9	25	
*NRAS*	25.0	10.4	71	
*KRAS*	16.6	12.6	15	

**Table 5:** Association between *RAS* Mutation AF and types of thyroid carcinomas.

Type of Thyroid Cancer	Average *RAS* AF (%) of Patients	Standard Deviation (SD)	n	*p*-Values
Follicular thyroid carcinoma (FTC)	30.8	12.3	5	0.104
Papillary Thyroid Carcinoma (PTC)	22.5	10.9	105	

Please note that the final surgical pathology of 1 patient with *TERT* and *RAS* mutations is unknown.

**Table 6:** *RAS* Mutation AF in *TERT* and *RAS*-positive vs. *RAS*-only thyroid nodules across PTC and FTC subtypes.

Groups	Average *RAS* AF (%) of Patients with *RAS + TERT* Co-Mutation	Average *RAS* AF (%) of Patients with *RAS* Mutation Only	Standard Deviation (SD)	n	*p*-Values
Subtype of FTC					
Widely invasive FTC	42.0	-	N/A	1	N/A *
Minimally invasive FTC	28.0	28.0	12.3	4	
Subtypes of PTC/NIFTP					
NIFTP	-	23.0	9.2	19	
FVPTC	-	24.1	10.0	44	
Invasive FVPTC	-	22.3	11.5	12	
Differentiated high-grade FVPTC	42.0	-	N/A	1	
Oncocytic	45.0	11.3	18.2	4	
Classical	-	35.0	N/A	1	
Solid/Trabecular	-	25.5	21.9	2	
Macrofollicular	-	19.0	14.1	3	
Well-differentiated thyroid carcinoma (not otherwise specified)	-	16.0	N/A	1	N/A *
Papillary microcarcinoma	-	10.0	N/A	1	
PTC in adenomatoid nodule	-	25.0	N/A	1	
Benign pathology					
Follicular nodular disease	-	12.3	11.2	6	N/A *
Follicular adenoma	-	17.8	9.9	6	
Oncocytic adenoma	-	21.0	11.4	3	

* Statistical analysis was not performed due to low sample size (<5) in a vast majority of the cell counts, which violates the assumptions of the Chi-square test. Two patients in the *TERT* and *RAS* group were excluded from Table 6 classification due to unavailable or inaccessible final surgical pathology for the *RAS*-positive nodule.

## Data Availability

Data supporting the findings of this study can be obtained from the corresponding author upon reasonable request. Public sharing of the data is restricted in accordance with the ethics approval agreement.

## Abbreviations

AF	Allele Frequency
CNA	Copy Number Alteration
CI	Confidence Interval
FTC	Follicular Thyroid Carcinoma
GEP	Gene Expression Profiling
NIFTP	Non-Invasive Follicular Thyroid Neoplasm with Papillary-like Nuclear Features
PTC	Papillary Thyroid Carcinoma
RAS	Rat Sarcoma Virus Oncogene Family
SD	Standard Deviation
TERT	Telomerase Reverse Transcriptase
